# How Social Networks May Influence Cancer Patients' Situated Identity and Illness-Related Behaviors

**DOI:** 10.3389/fpubh.2018.00240

**Published:** 2018-09-04

**Authors:** Eric C. Jones, Martin Storksdieck, Maria L. Rangel

**Affiliations:** ^1^School of Public Health, University of Texas Health Science Center at Houston, Houston, TX, United States; ^2^Center for Research on Lifelong STEM Learning, Oregon State University, Corvallis, OR, United States

**Keywords:** social networks, cultural characteristics, cancer prevention, decision making, cancer survivorship, social support in cancer

## Abstract

Little research is currently available that captures variation in the degree to which individuals who have, or had cancer in the past (but are in remission) integrate their cancer experience into their sense of self or their cancer-associated identity. Such research should cover how those identities shape personal narratives within existing or new social networks so that, ultimately, we understand the implications for treatment choices and health outcomes. Particularly understudied are the social factors influencing the incorporation of cancer into identity, learning, and behavior. Social network analysis captures specific relationships, what they offer, and the structure or constellation of these relationships around someone who has cancer or has had cancer. Some studies point to potential cultural differences in ethnic or social groups in how social influences on the cancer experience play out in terms of individual coping strategies. In some populations, social cohesion or tight networks are common and of particular importance to individuals and include social institutions like church communities. Social status might also generate social pressures not typically noticed or experienced by other groups. We will discuss how social network analysis can be used to elucidate these factors and, conversely, how the specific context of cancer diagnosis can be used through social network analysis to better understand the role of community in helping individuals address situations of severe adversity.

## Introduction

This mini review tries to shed light on how social network analysis helps us understand the impact of social support and social interactions on patients in the cancer care continuum—from cancer prevention to survivorship. We evaluate the strength of various social factors in predicting how people with cancer develop their identities and related learning behaviors in response to having cancer, and primarily how these identities might be shaped by, or are shaping the social environment of cancer survivors. Pertinent social factors that intersect with different kinds of social status (e.g., gender, ethnicity) include family, survivor support groups, health care providers, friends/coworkers/employers/other acquaintances and, increasingly, people in online social networks. Relationships with these different roles produce social constraints, opportunities, and types of connections and resources. We are exploring how the constellations of pertinent social factors might interact in creating new identities for some people; identities that are adapted to this potentially life-threatening situation of cancer.

This manuscript covers social network analysis and cancer, and it focuses on how interpersonal relations might impact the ways in which people form new identities when they have cancer. Social network analysis has been employed in several fields of study related to the practice of medicine with cancer, including diffusion of treatment practices (1–3), leadership in cancer research networks (4, 5), community coalitions to reduce cancer disparities (6, 7), and ways in which hospitals themselves are connected through their cancer patients (8), and the enrollment of cancer patients in health insurance (9). In the rest of this review, we do not consider those areas of social network analysis—rather only interpersonal relations of people with cancer or caregivers.

## Components of an integrative model of social networks and cancer survivorship

**Figure 1:**
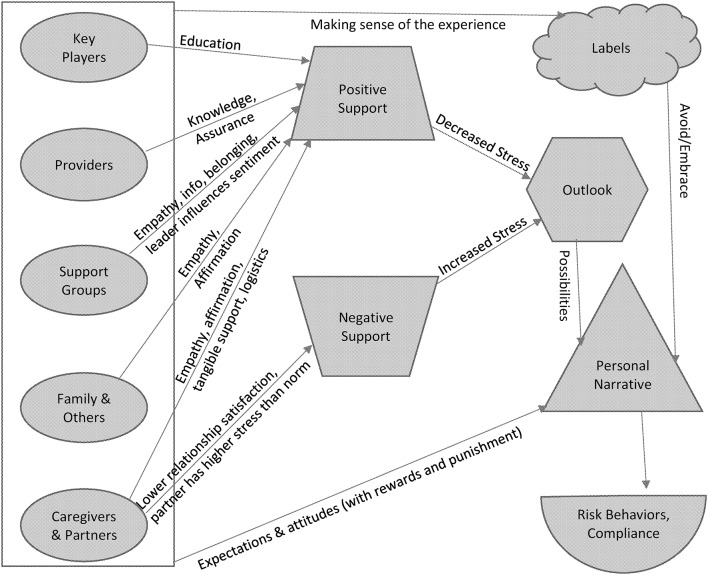
Conceptual Model of Social Influences on Integrating Cancer Experiences into a Personal Narrative or Identity.

While individuals' own personal suites of dread, acceptance, fortitude, resignation, anticipation, hope, and numbness shepherd their expectations, regret, and resolve, these personal suites are moderately weak filters for finalizing the valences of all of the outside or social influences—when we are vulnerable due to our health, we also vulnerable to many other things, including the words and deeds of those with intimate knowledge of our situation. To examine the various pathways through which these social influences cumulatively operate, we present a graphical model of social influences on refiguring one's personal narrative with cancer (see Figure 1).

Our conceptualization of the findings from existing research on social influences resides in Figure 1—a conceptual graphic modeling of the dynamics and mechanisms of social influence on people with cancer. We review both personal networks and whole networks comprised of individuals who bring their relationships to bear upon an individual or individuals who may be at any stage of addressing their health status impacted by cancer. In most basic form, these networks are based on dyadic relationships, or the specification by the researcher of some type of tie between any two people of interest. These dyads add up to show patterns of content and structure of general or specific social worlds of people with cancer. In Figure 1, we have tried to capture the following dynamics or characteristics:

- Social aspects of telling or not telling others, based on their attitudes, expectations, support, and labels
- Specific social constellations
  - structure of a network, such as subgroups, high density, or sparse relations
  - homogeneity and heterogeneity of network member characteristics
  - specific categories of types of relationships—spouse, adult children, children, support groups, media, providers, friends, coworkers, bosses, neighbors, social groups, clubs, acquaintances, online networks
- Interaction of individual's situation and personality with these social constellations
- Consequences for risk-related behaviors, compliance, and wellbeing

People influence those with cancer through support, through making sense of the cancer/treatment/risk via labels, plus through expressing attitudes and expectations for those that have cancer. Arrows from the ovals in Figure 1 to the positive and negative support are based on empirical studies, as is the connection between Personal Narrative and the outcomes of Risk Behaviors and Compliance in the lower right. The rest of the model in Figure 1 is hypothesized. The following sections basically concern existing research on the arrows from the ovals to positive and negative support. While research has not been done on other aspects of the model, we think it is important to present in a larger framework rather than in isolation.

## Key players for cancer education

Peer-based interventions can be effective and important in creating an environment that makes healthy choices easier (10). Special kinds of peers include key players or well-connected people in community networks. In the area of cancer prevention, peer education has promoted the consumption of fruits and vegetables to reduce cancer risk (11). That project used social network analysis to detect cliques—or sets of connected individuals—among work groups in public institutions in two Arizona cities. Then their peer educators were chosen based on having a high peer index—a combination of the following network measures: serving as a bridge between others (i.e., betweenness centrality), number of ties (i.e., degree centrality) which suggests social prominence, and the average strength of each of the ties with the people named by that interviewee (they could each name up to eight). Finally, the peer education occurred within each of the cliques to take advantage of social reinforcement, and the program generally resulted in increased vegetable intake and improved attitudes toward fruit and vegetables at 6-months follow up.

In another study of key players for cancer prevention education, a structured snowball sampling technique and social network analysis helped discover the key players over 50 years of age in a community to examine their roles in improving the coverage of colorectal cancer screening (12). Scholars then interviewed the key players to understand the salience of occupational and educational status and personal attributes like communication skills and resourcefulness. Although the study captured the characteristics and behaviors of structurally key people, it did not systematically examine the impact of the network or these key players on screening or health outcomes. A study of people promoting colorectal cancer screening by sending emails to their personal networks found that 3 in 4 people were willing to send a message to their networks as long as they could revise the message to fit their voice (13). Also, attendance at cancer education seminars can be used to increase the size of people's networks (14).

## Support from family, friends, and others

Communication between family members about hereditary risks and genetic testing can impact other family members in cancer screening and other forms of cancer prevention. A study of a form of hereditary cancer found that people with these mutations communicated about genetic test results with more family members than did those who did not have mutations, supposedly because of higher saliency in their message to family members (15). The latter (no mutations) communicated mainly with people whose advice they tend to use in general (despite also listing more total *family* members than did mutation-positive respondents). Otherwise, respondents overall were less likely to talk about risk to family members younger than themselves, and more likely to share thoughts about risk with those who are close and/or provide emotional support.

Support from friends and family was important across 11 qualitative studies concerning whether African-American women decide to undergo cervical cancer screening (16). On the other hand, lack of information on family history was a major barrier for Hasidic women to screen for hereditary breast and ovarian cancer (17). Orthodox Jews look not only to opinions of family on getting screened for hereditary breast and ovarian cancer, but friends, community members and particularly faith-based leaders (18). Social capital (not social network analysis *per se*) has been championed to promote screening among African American church members due to a three-fold increase in prostrate screening for every point of increase in their community participation scale (19), perhaps in part due to lack of trust in mainstream medical information transmission.

Having a family member with cancer increases individuals' risk assessment for contracting cancer themselves and expands the range of perceived for risk factors. However, exposure to family with cancer does not change one's opinion of their own ability to control cancer or reduce one's risks (20). That study of foreign-born Asian Americans potentially suggests a kind of fatalism in this population that renders them somewhat immune to certain kinds of social influences. In another study that did not rely on dyadic network data, investigators found that having children and good information or education about treatment increased the odds of people following correctly their oral chemotherapy treatment (21), presumably due to higher confidence about the treatment and higher motivation to be alive for their kids.

Direct influences on cancer-related behaviors also constitute a focus for social network analysis. To capture perceived support for healthy eating and active living from people close to them in a study of 70 Latina breast cancer survivors, scholars used Cohen's Social Network Index–an interview about the number and type of people with whom you interact to measure size and diversity of one's personal network—and they also used a modified General Social Survey (GSS) networks module that asks about people with whom they discuss important matters (22). Family members were key to healthy eating behaviors for these women, but they were also cited as a barrier to healthy eating for half of the women. Their networks included very few people from work, education or volunteering, and mainly family—especially their children—plus friends and neighbors. Interestingly, network diversity was not associated with the interviewee's healthy eating. The lack of impact of relationship diversity—where diversity would increase exposure to different attitudes and behaviors—may result from the prominence of mechanisms cited by the authors for influencing dietary choices. These influences involved information consumption via descriptive norms (from the field of social psychology) or social selection (from the field of sociology) based on the perceived frequency of those behaviors; social approval though injunctive norms (from social psychology) or social causation (from sociology) based on whether people are seen as doing the morally or culturally correct thing; and finally social transmission of behaviors in which variation exists regarding the settings and communication media in which people experience either of these types of norms.

Although not asking respondents to list specific people, researchers found that being less isolated and receiving various types of social support were associated with higher quality of life measures for breast cancer survivors (23). Highest quality of life was for women naming at least 15 close family and friends, any church participation, any volunteering, and being married. Similarly, based on social support questions in a survey rather than on dyadic social network relations about specific people in one's life, scholars found that African–American women accepted sympathy and found greater support in church more so than did Caucasian women with breast cancer (24).

## Online support groups

In an online smoking cessation group for women with a site moderator participating in some of the discussions, a core group of participants emerged who were connected through highly connected hubs (25). This core was not connected to others who were very sparsely connected to other conversations on the discussion board and mainly connected to these other conversations and participants only through interaction with the site moderator. Interestingly, for those smokefree for less than a year, there was a positive correlation with eigenvector centrality that measures whether you interacting with people who interact with a lot of other people, while it was a negative association with eigenvector centrality for those who were smoke free for more than a year. Other authors believe this is because social network site participants who quit recently were more likely to respond quickly to posts (26), although it is worth considering the complimentary thesis that once participants have been successful at quitting smoking over the long term they feel less need to participate.

One small online breast cancer survivors group interacting via a listserv in Scandinavia focused on using storytelling to generate exchanges of information and experiences, and researchers found that all participants experienced social isolation or loneliness and that all also talked about social support or provided encouragement (27). All but one of the 15 also discussed information giving and seeking. It wasn't the anonymity, but rather the lack of physical contact in the listserv that allowed the women to broach very difficult topics and thus created new trust with each other and a general feeling of being less isolated.

In an online network—this one focused on melanoma, and specifically on a network of interactions about the chemotherapy—a distinct caregiver group that had a core doctor and nurse and peripheral patients emerged separately from a distinct patient group that had peripheral caregivers (28). The two groups nonetheless had connections between them—but mostly through a set of bridging individuals since most people were not participating in both groups. The facilitator in that online melanoma network was the most frequent participant and also the greatest bridge between various people's conversations, as in the online smoke-free network described earlier.

## Caregivers

The toll of cancer on those who provide support for survivors has generated several network studies. Considering ethnicity, Caucasians were least likely to have positive views about their experience giving care to cancer patients than were other ethnicities in a study of 111 informal caregivers (29). In a social network analysis study of caregivers and patients in palliative care, researchers gathered data on people in one's life, as well as the relationships between each of the people named by the interviewee (30). Interview questions focused on the patient, the family caregivers and the professional caregiver—specifically covering the contacts between people in the network, an evaluation of the services provided, and gaps and continuity in care. Some of the questions asked in the study can be classified as cognitive network questions—or an evaluation by a network member of the interactions between other network members. This approach can be useful for examining the degree to which there is overlap and diversity in perspectives within a network.

## Providers

Despite the increased responsibility and pressure that comes with it, many cancer patients are feeling augmented agency in many settings in the last decade or so (31). This agency includes health care providers and professionals describing options, making information available to patients, and acknowledging that the decision makers really are the patients (32). This personal agency stems from how health care providers structure the decision-making situation. Some are sharing information and promoting decisions in ways that promote successful outcomes and that increase their bottom line financially (and/or reduce their risk to financial loss), but many are reluctant to unduly influence the decision of the patient. Here is where even greater agency can come in—patients finding ways to get doctors to say what they would do for themselves or their spouse in any particular situation (32). These patients don't want the full responsibility for decision-making, but want experts to give their absolute best guess as to what would be right for them. Thus, health care providers and other health professionals are gatekeepers of information who are co-creating with patients certain kinds of social influences that can both support and constrain the patient and those who are helping the patient discuss decisions. However, the pressures to act quickly upon a cancer diagnosis might not come from medical teams but from one's own family and friends, and even coworkers and acquaintances.

## Impersonal relations

Online or internet interactions not based on a listserv or online support group can sometimes represent a less personal set of interactions, but they undoubtedly play a role in how people decide to approach decisions about cancer treatment. In a social network analysis study on Twitter and cancer messages, retweeting about breast cancer was predicted by in-degree centrality or the number of followers, betweenness centrality, and closeness centrality—especially the latter—but only around 7% of messages were retweeted (33). These scholars pose the interesting idea that the common conceptualization of opinion leaders as always followed by other consumers of information may need revision in order to capture the kinds of interactions involved in consuming and reproducing and revising information and in order to understand the diversity of roles in online dissemination that do not necessary fit a unidirectional flow or a hierarchical structure. Since impersonal relations are not typically pointed personally at an individual cancer survivor—with the exception of celebrities or public figures—or from them to other specific people, it is really more up to the survivor to make decisions about how to engage such interactions. Thus, in Figure 1, we anticipate that the two arrows from the outline of the box on the left (i.e., not connected to specific ovals/actors on the left) labeled “Making sense of the experience” and “Expectations and attitudes” are the social mechanisms by which impersonal relations are typically going to impact a person's personal narrative and identity around cancer survivorship.

## Summary and conclusion

Individuals who seek emotional support directly from others are considered empathy seekers and providers; those who seek and discuss medical information related to their specific disease are information seekers and providers; and those who begin to support a community of cancer patients and survivors are advocates. People may do none, any or all of these at any given time. Conversely, some individuals may retreat from social connections and keep their diagnosis private, because they don't like sharing personal details with others, because they see themselves as a burden to others if they did, or because sharing would force them to acknowledge the disease. Thus, even without informing others about their cancer, a person is reacting to general and even specific social factors. As one deals with cancer, they have the choice of staying within their own network, potentially expanding or contracting it, or even totally changing it for the purpose of dealing with the disease. For minorities, this varies with the contexts in which their minority status is activated or acted upon by others.

Social network analysis captures specific relationships, what they offer, and the structure or constellation of these relationships around someone who has cancer or has had cancer. The research involving social networks and cancer have illuminated a great deal regarding treatment decision making, cancer prevention, and other aspects of interpersonal relationships related to cancer information, behaviors and outcomes. In the other direction, cancer duration, comorbidity, and primary treatment types likely also have independent impacts on networks. Finally, these social influences do not occur independently of one another.

In addition to the ways that a cancer patient's personality and internal motivations guide their social interactions, social networks mediate behaviors though structural variation in one's network and through the degree of diversity of people with whom cancer patients interact. Homogeneity and heterogeneity are extremely important social factors for populations that are considered minorities. Additionally, there are often multiple different influences from the same person since many of our relationships are multi-faceted, the influences come and go, and they can be cumulative. The ultimate goal of this vein of social science research is to develop advice that helps minority cancer patients develop individualized support structures that help improve outcomes.

## Author contributions

EJ and MS conceptualized the manuscript. MR created Figure 1 and integrated the concepts into Figure 1. EJ, MS, and MR all reviewed appropriate literature. EJ created the main draft, and additional comments and writing undertaken by MS and MR.

### Conflict of interest statement

The authors declare that the research was conducted in the absence of any commercial or financial relationships that could be construed as a potential conflict of interest.
